# Feasibility study of ultrasound-guided percutaneous laser discectomy for cervical radicular pain

**DOI:** 10.1038/s41598-022-17627-9

**Published:** 2022-08-02

**Authors:** Rong Hu, Xiaolei Zhu, Yi Zhou, Jianping Zhang, Dong Huang, Haocheng Zhou

**Affiliations:** 1 Department of Pain, The Third Xiangya Hospital and Institute of Pain Medicine, Central South University, Changsha, China; 2 Hunan Key Laboratory of Brain Homeostasis, Central South University, Changsha, China

**Keywords:** Chronic pain, Pain management, Neurodegenerative diseases, Neuropathic pain

## Abstract

Percutaneous laser discectomy is one common and effective treatment for cervical radicular pain. Currently, the surgery is performed with blind cannulation technique, mainly relies on the experience of surgeon. However, it still remains unsafe and difficult to reach the target. As an alternative, ultrasound-guided cannulation provides visualization of important structures, thus increasing the precision and safety. The primary goal of this study is to report the detail of the ultrasound-guided technique in the percutaneous laser cervical discectomy. The secondary purpose is to evaluate the feasibility of the novel therapy. This is a single center, feasibility study conducted in one teaching hospital. Thirteen intervertebral discs in 9 patients presented with cervical radicular pain. Accuracy of the cannulation with ultrasonic guidance was confirmed by the anterior–posterior and lateral view of fluoroscopy. We compared the pain severity pre- and post-treatment with Visual Analogue Score (VAS), and functional improvement was assessed with the modified Macnab Criteria and Neck Disability Index (NDI) respectively. Ultrasonic short-axis was used to scan the cervical nerve root, and its transition was used to identify the distinct intervertebral space. Following the recognition of targeted cervical level, the ultrasound probe was moved medially for the visualization of the surface of the cervical vertebrae. In plane cannulation was then applied to avoid the injury of the vessels. The location of cannula was confirmed by the fluoroscopic imaging. Low-power laser was set for the cervical disc ablation in this cohort. The majority of the surgical sites maintained in the C5/6 level (38%), and 31% for the C6/7 level respectively. Despite the distinct cervical level, the tip of needle was properly placed near by the targeted intervertebral disc in all participants, which was confirmed by the imaging of fluoroscopy. We did not observe any obvious complications during the procedure. The mean VAS decreased from 7.6 ± 1.1 to 2.3 ± 2.7 one month after discharge, and 2.1 ± 2.6 at the last follow-up (median duration of nine months). All patients reported significant improvement of NDI up to last follow-up (p = 0.011). Meanwhile, the good to excellent rate was reported in 8 of 9 patients (89%) according to the modified Macnab Criteria. The finding of this feasibility assessment indicates the ultrasound-based cannulation technique is capable of guiding the cannulation for the percutaneous laser discectomy. It may facilitate identifying the corresponding site of cervical intervertebral disc and prevent the damage of vessel.

## Introduction

Cervical radicular pain is caused the degeneration of intervertebral discs, characterized by the localized, referred, or radiated pain in the affected dermatome^1^. The overall estimated one-year prevalence of cervical pain is about 37.2%, ranged from 16.7 to 75.1% for the entire adult population^2^. Conservative therapy remains the first option in most patients for initial management of cervical disc herniation, including non-steroidal anti-inflammatory drug, muscle relaxants, and physiotherapy^3^. The symptoms may be attenuated in about 60–80% patients 6 weeks after the initial onset of pain^4^. Surgical intervention may be considered when conservative therapies fail to control pain, especially for those with severe radiculopathy or myelopathy^5^.

Percutaneous laser discectomy (PLD) is one minimally invasive procedure, which can be used for the treatment of cervical, thoracic and lumbar disc herniation^5–7^. The enduring therapeutic effect of the minimally invasive surgery maintained in 76% patients who suffered cervical radicular pain^8^. Multiple modes of action may contribute to the rapid improvement of surgery, including vaporization effect, shrinkage of collagen, decompression effect, denervation, and anti-inflammatory effect^9^. The superiority of percutaneous technique over traditional open surgery remains controversy^10^, however, one obvious advantage of the minimally-invasive therapy is the reduction of structural damage and postoperative pain.

Although the tissue damage is limited due to the minimally invasive incision, we can still not ignore the risk of structural injury in the cervical region (e.g., carotid and vertebral artery, jugular vein, trachea, esophagus, thyroid, and nerves). The complication rate was reported in about 1.0% patient, including hematoma, structural lesions, infection, thromboembolic and neurological complications^10,11^. Thus, the percutaneous technique should be only conducted by the experienced surgeons, to achieve better clinical safety.

Under the guidance of ultrasound imaging, physician can visualize the cervical structures with significant improvement of resolution. Meanwhile, the use of ultrasound-guided technique has been applied for management of axial and peripheral pain in the cervical region^12,13^. Compared with conventional cervical nerve block, the puncturing entry for cervical intervertebral space is relatively closed to the middle line, leading greater risk of injury to the internal jugular vein and carotid artery. To our knowledge, few study has focused the role of ultrasound-guided detail for the PLD.

In this study, we aimed to provide our experience of ultrasound-guided PLD in the pain management of cervical radiculopathy, and to assess the feasibility of this novel treatment.

## Materials and methods

### Study design and participants

This was a retrospective, single center, observational study, conducted by the Pain Department of The Third Xiangya Hospital. The medical recordings of nine patients with cervical radicular pain, who underwent ultrasound-guided PLD were retrospectively reviewed. The inclusion criteria included as follow: (1) patients who were diagnosed with cervical radicular pain according to history, physical examination, and imaging test; (2) pain severity was reported above 4 of 10 Visual Analog Scale (VAS) after conservative treatment; (3) patients who were willing to undertake the mini-invasive procedure. Exclusion criteria included: (1) cervical spinal instability; (2) severe spinal stenosis; (3) calcification of annulus fibrosus; (4) ossification of the posterior longitudinal ligament.

All nine patients were informed about the detail of interventional treatment prior to procedure, and all of them consented to undertake the surgery and signed the informed consent. The study was conducted in accordance with the Declaration of Helsinki, and the protocol of this study was approved by the Ethics Committee of The Third Xiangya Hospital, Central South University (2016-S240).

### Preoperative preparation

Preoperative assessment is necessary to determine the targeted cervical level, and the symptomatic discs can be confirmed by the MRI prior to procedure. Previous study supports the use of provocation discography in cervical perfusion^14^. However, it remains unclear the role of discography in PLD. Thus, discography is not routinely performed before surgery according to the guideline in our center^15^. Patient was required not to drink or eat on the day of procedure, and sedative drug is administrated during the surgery if needed. One single shot prophylactic treatment of cefuroxime was applied intravenously 30 min prior to the surgery. Local anesthesia is performed with a solution of 1% lidocaine infiltration into the skin and subcutaneous tissue.

### Ultrasound guidance

**Figure 1:**
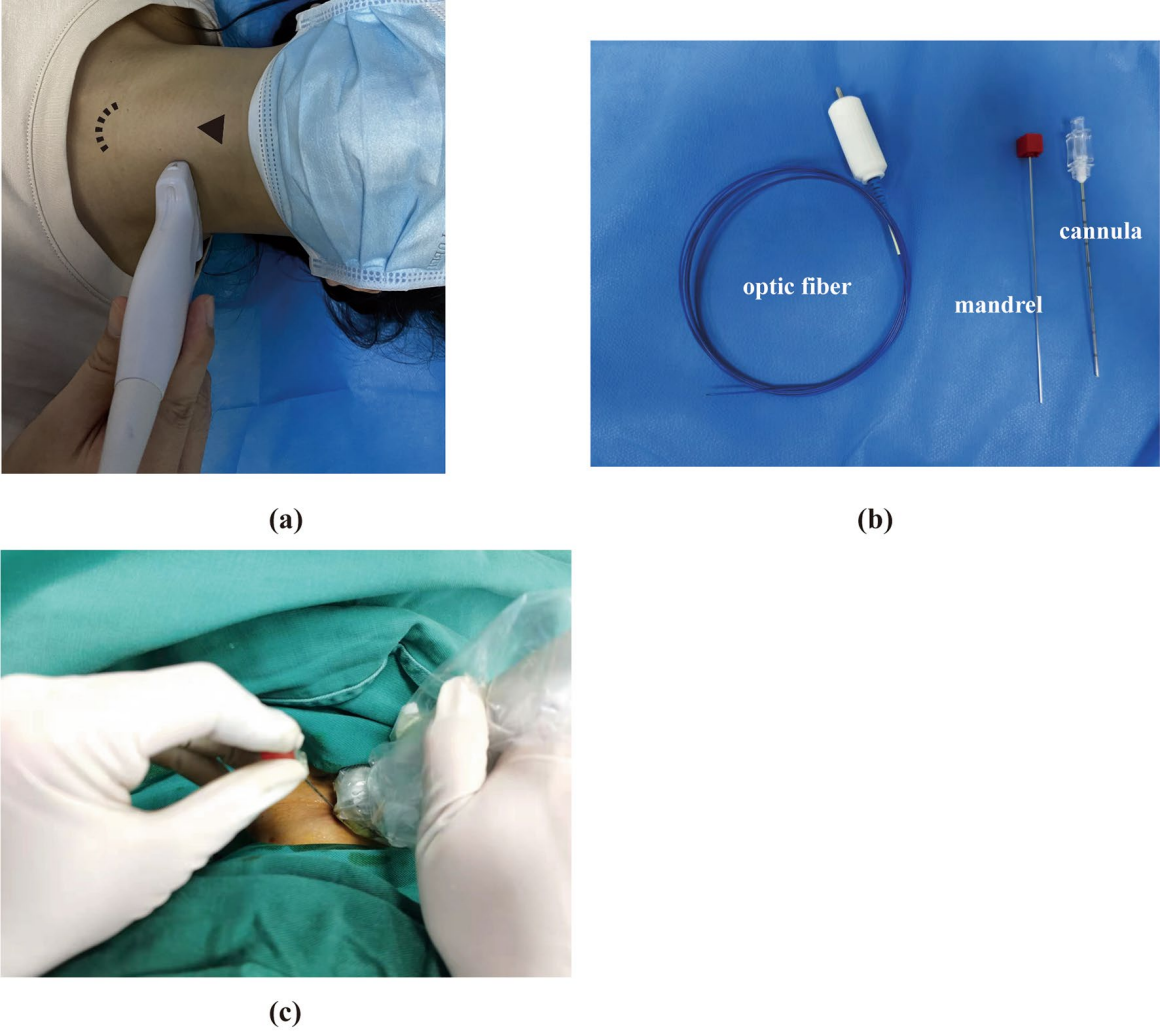
Surgical device of PLD. (**a**) Schematic of ultrasound probe placement for initial scanning. The probe is placed between the sternal notch (dash line) and superior pole of the thyroid cartilage (triangle) in one short-axis plane. (**b**) Percutaneous cannulation and laser equipment. (**c**) In-plane ultrasound-guided technique of needle insertion.

The patient is placed in a supine position with shoulder slightly elevated to provide better visualization of lower cervical disc space (C6/C7). One high-frequency linear probe (Fujifilm, SONOSITE, United States) is used to scan the cervical region (Fig. 1a), and the depth of scanning is set 4–5 cm approximately. One 19-G cannula with an internal mandrel was inserted under the guidance of ultrasound, the device of cannulation is shown in the Fig. 1b. The in-plane ultrasound guidance is used to insert the cannula (Fig. 1c).

**Figure 2:**
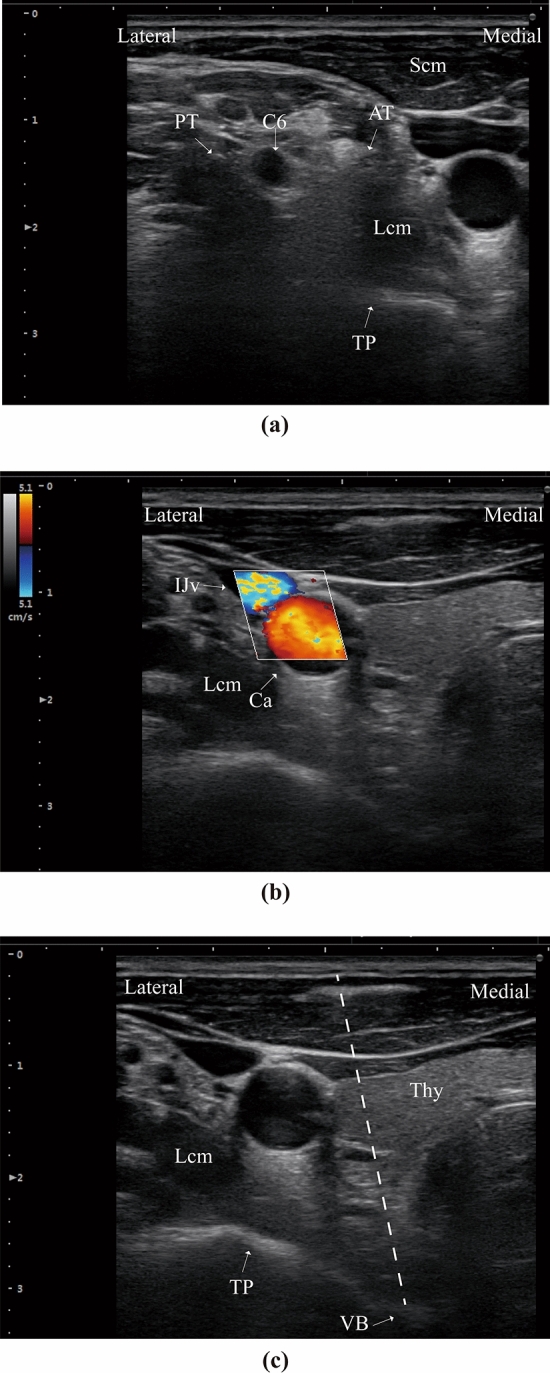
Guidance of C5/6 intervertebral space. (**a**) Characteristic U-type shape of the tubercle at C6 nerve root level. (**b**) Identification of carotid artery and jugular vein with color flow doppler. (**c**) Design of puncturing path (dash line) to the anterior surface of C6 vertebral body. *Scm* sternocleidomastoid muscle, *Lcm* longus colli muscle, *AP* anterior tubercle, *PT* posterior tubercle, *TP* sixth transverse process, *VB* sixth vertebral body, *Thy* thyroid, *C6* sixth cervical nerve root, *IJv* internal jugular vein, *Ca* carotid artery.

It is important to identify the cervical structures before puncturing. The detail of sonoanatomy at different cervical levels is described as previously^16^. The first step is to identify the transverse process of C6 and its corresponding tubercles. Specifically, the ultrasound probe is placed in an axial position at the level of the cricoid cartilage. The anterior (Chassaignac tubercle) and posterior tubercle is easily identified by the characteristic U-type shape, as shown in Fig. 2a. To avoid the injury of internal jugular vein and carotid artery (Fig. 2b), we move the probe towards the middle line to scan the space of cannulation. In Fig. 2c, the path between puncturing entry and the anterior surface of vertebral body is designed to perform the cannulation of C5/6 disc.

**Figure 3:**
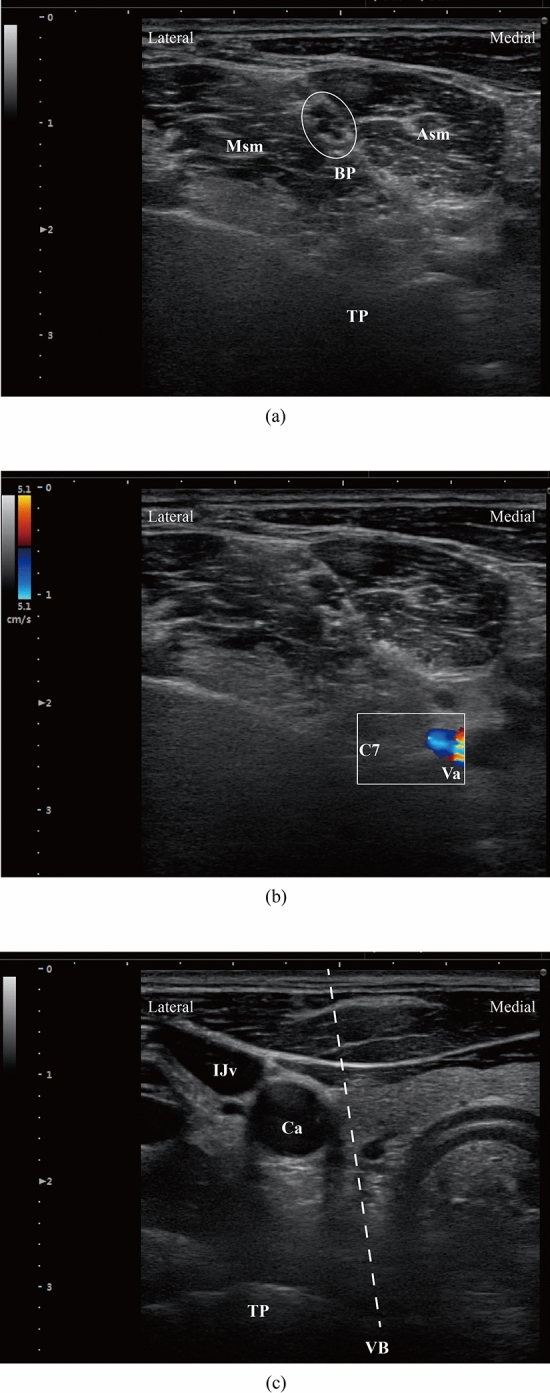
Ultrasonic imaging for C6/7-disc level. (**a**) Transverse process is straight at C7 level, the brachial plexus can be recognized by its grape-like shape. (**b**) C7 nerve root is slightly lateral to the vertebral artery in the short-axis ultrasound image. (**c**) Cannulation access of C6/7 intervertebral disc space. *Asm* anterior scalene muscle, *Msm* middle scalene muscle, *TP* seventh transverse process, *VB* seventh vertebral body, *BP* brachial plexus, *C7* seventh cervical nerve root, *Va* vertebral artery, *IJv* internal jugular vein, *Ca* carotid artery.

Following the initial identification of C6 vertebral body, we can gradually scan the C7 levels by moving the probe inferiorly. One can easily distinguish the C7 from other cervical levels due to its straight transverse process (Fig. 3a). When performing the cannulation at this level, we must be careful enough to avoid to insert the needle into the vertebral artery (Fig. 3b). The pathway of C6/7 interverbal disc is given in the Fig. 3c.

**Figure 4:**
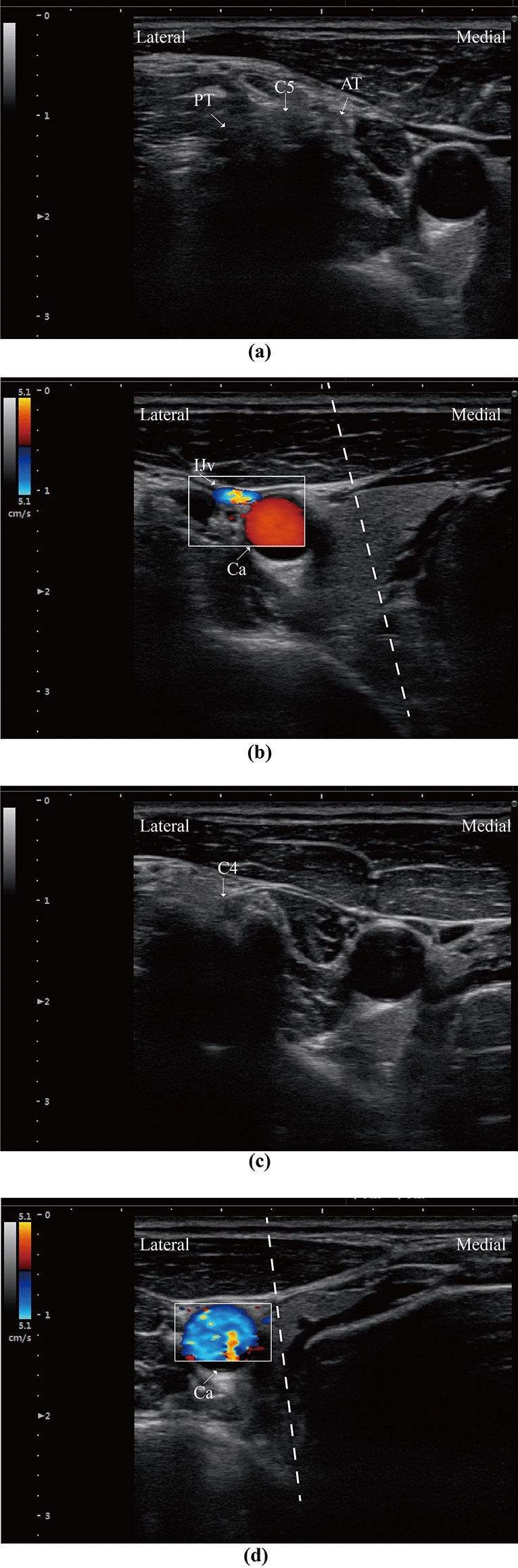
Ultrasound-guided cannulation for upper cervical intervertebral space. (**a**,**b**) Imaging scan of C5 level, and placement of cannula into the C4/5 disc. (**c**) Sonography of C4 cervical nerve root. (**d**) Cannulation pathway for C3/4 intervertebral disc.

Similarly, the upper cervical level can be identified subsequently when we place the probe cephalad. The intertubercle sulcus becomes vertical at C5 level (Fig. 4a), and smooth and superficial for C4 nerve root (Fig. 4c). The transition of transverse process and puncturing access at C4 and C5 level is given in the Fig. 4, respectively.

### Low-power discectomy

**Figure 5:**
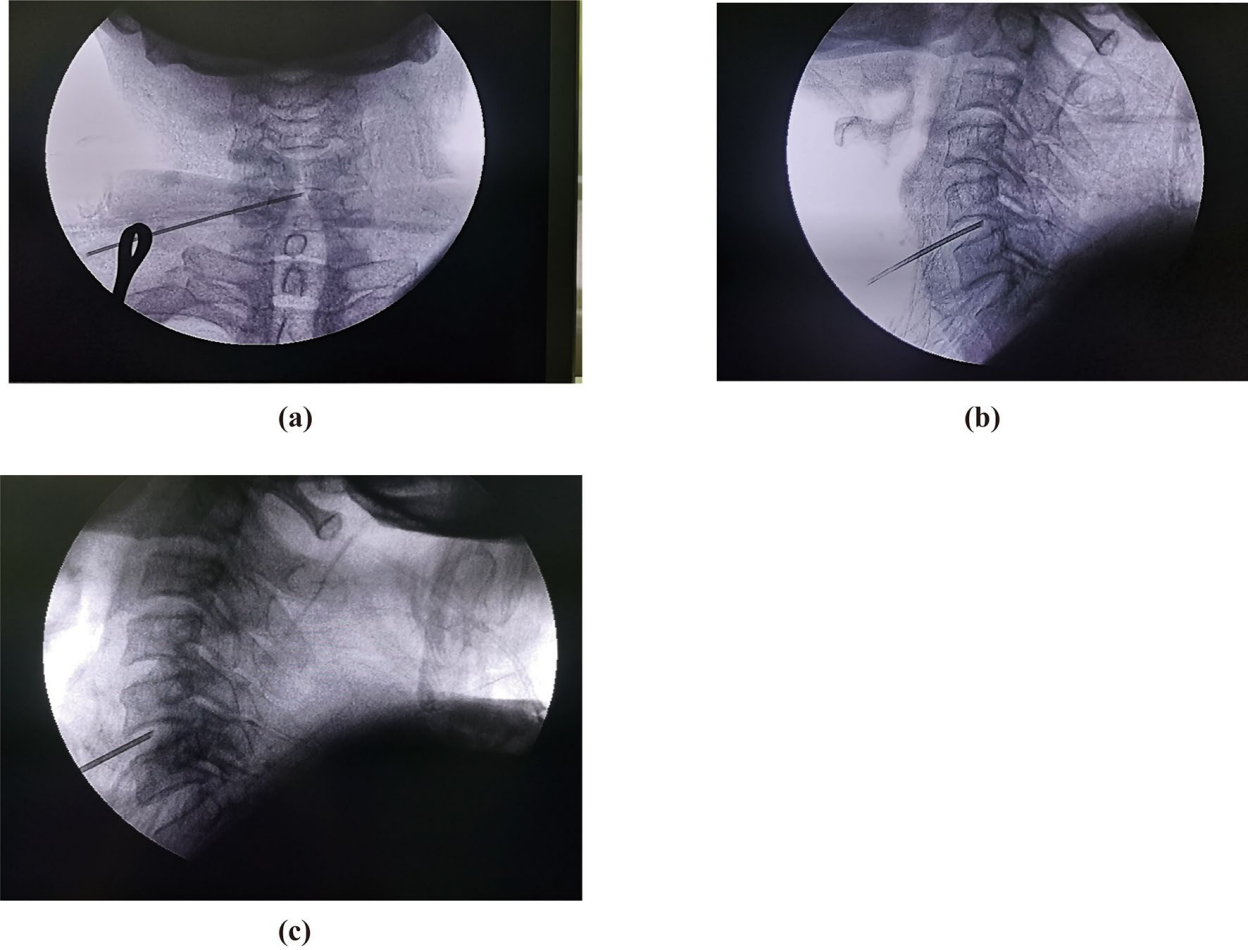
Fluoroscopic imaging of cannula position. (**a**) Anterior–posterior and (**b**) lateral view of cannulation at initial surgical site. (**c**) The second site of laser decompression is located in the middle of intervertebral disc, confirmed by the lateral view of fluoroscopy.

Upon the cannula is placed in the interverbal space and stable, we can insert the cannula gradually under the guidance of fluoroscopy with a C-arm unit. The cannula tip should be placed at the distal third segment of disc, confirmed by the lateral fluoroscopic view (Fig. 5a). Meanwhile, the tip is slightly beyond the middle line in the anterior–posterior view (Fig. 5b). One optic fiber (200-μm diameter) is inserted through the cannula, and the distal ending of fiber is connected to one laser generator (Alaude-01; Keheng, Heilongjiang, China). We apply low power laser (2 W, pulse width of 1 s) in the procedure. The lower power laser may prevent potential tissue damage by the heat radiation, compared with conventional laser therapy (over 10 W). During the laser decompression, one small volume of saline (1–2 ml) is injected into the disc, and we can see several air bubbles up through the cannula due to the effect of vaporization. The total output of laser power is set about 150–200 J for the first cycle. Then we pull out the cannula and fiber to conduct the second cycle, the tip of cannula should be positioned at the middle of the disc during the second cycle (Fig. 5c). The total out of laser was no more than 350 J for one cervical disc to avoid exceeding degenerated changes.

### Postoperative care

Flurbiprofen (100 mg per day) is routinely administrated in the next two days after surgery to provide analgesic and anti-inflammatory effect. Patient is asked to stay in bed for two days at least, and a rigid neck collar must be wearing in the following month after discharge due to the potential instability of cervical spine.

### Clinical outcome

VAS is used to assess the pain severity in patients, rating from 0 (“pain free”) to 10 (“worst pain imaginable”) pre- and post-treatment. The modified Macnab Criteria and Neck Disability Index (NDI) were used to examine the pain relief and functional improvement after PLD therapy^17^. The routine follow-up is scheduled at 1-, 3- and 6-month after discharge. All patients finished the first two follow-ups by one independent researcher (X.Z), who did not conduct the procedure. The patients were asked to score their current pain during each follow-up. All patients were followed at least 5 months after PLD surgery, with a median duration of nine months (ranging from 5 to 13 months).

### Statistical analysis

Descriptive analysis was used to present the clinical features of enrolled patients. Pain scores were presented as mean ± standard deviation, and the NDI values were given as median with its range respectively. The Shapiro–Wilk testing was applied to determine the normality value of each variable. Since data did not correspond to a Gaussian distribution, we compared pain severity and functional index between baseline and different follow-up timepoint using the nonparametric one-way ANOVA with repeated measures (Friedman test). Statistical significance was assumed for P value under 0.05.

## Results

Our patients include two females and seven males, ranging in age from 41 to 72 years old (mean 60, SD 11). The median duration of disease was three months. All patients presented with moderate to severe radicular pain, with mean VAS of 7.6 ± 1.1 at admission. The functional score assessed by the NDI ranged 11–76% (median, 40%) before the minimally-invasive therapy.

The following cervical intervertebral discs were ablated: C3–4, one out of thirteen (7.7%); C4–5, three out of thirteen (23.1%); five discs at C5–6 level (38.4%); and four for the C6–7 segment respectively. Six of nine patients (67%) underwent single cervical disc ablation, and two for dual disc level. Only one patient was treated with three consecutive intervertebral space (C3–6).

Before the initial exposure of the fluoroscopy, the cannulation was properly placed at the anterior surface of the cervical vertebrae in all patients, with the tip of needle 1–2 cm medial to the Luschka joint in the anterior–posterior view. None obvious adverse events (e.g., headache, dysphagia, hemorrhage, infection, or cerebrospinal fluid leak) were observed neither during the surgery nor post-treatment.

**Figure 6:**
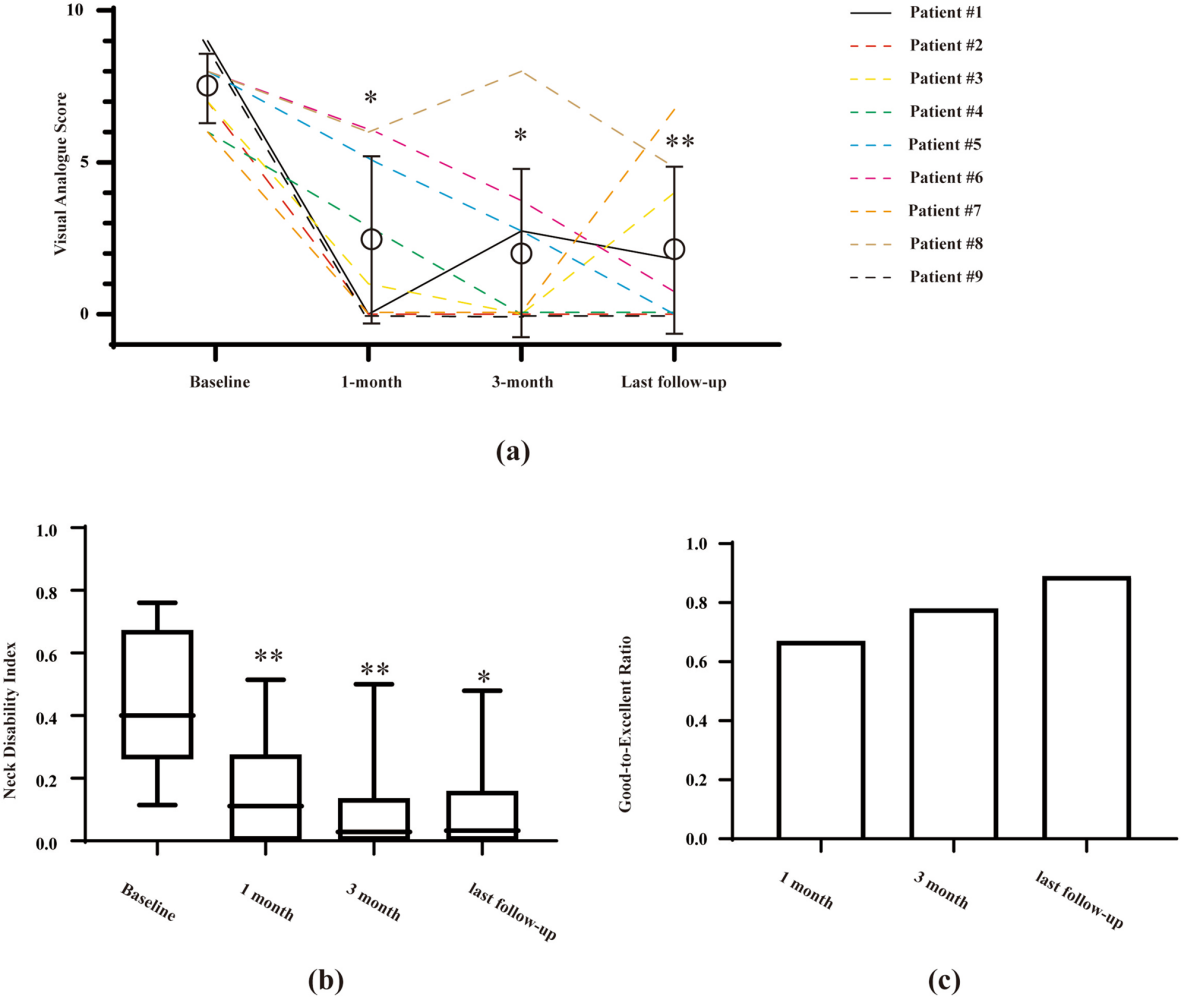
Comparisons of clinical outcomes pre- and post- PLD therapy. (**a**) Pain severity assessed by the VAS at 1-, 3-month, and last follow-up. Each line indicated one individual patient respectively across different follow-up points. Pain scores were compared between baseline and each follow-up time point, with Friedman’s non-parametric test and one-way ANOVA. *p < 0.05, **p < 0.01. (**b**) Significant functional improvement was achieved by the ultrasound-guided PLD. The NDI data is plotted as median with its range. Friedman’s non-parametric test and one-way ANOVA. *p < 0.05, **p < 0.01. (**c**) The modified Macnab Criteria collected at each time point.

To evaluate the pain improvement, we compared the VAS scores pre- and post-treatment in Fig. 6. a. Four patients (44%) reported total pain free at the 1-month follow-up, and 56% for the 3-month interview respectively. The average VAS decreased from 7.6 ± 1.1 to 2.3 ± 2.7 one month after discharge, and 2.1 ± 2.6 at the last follow-up (Fig. 6a). The overall response rate was 67% at last follow-up, with pain relief over 50% compared with baseline. Only one patient reported recurrent and intolerant neck pain (VAS: 7) with fatigue at last follow-up, and two with moderate pain (VAS: 4 and 5). In addition to pain severity, significant functional improvement was achieved since the first one-month follow-up, as demonstrated by the reduction of NDI (p = 0.008). The sustained improvement was reported at the 3-month (p = 0.004) and last follow-up (p = 0.011), as shown in Fig. 6b. Meanwhile, the good-to-excellent rate that evaluated by the Modified McNabb Criteria, gradually increased from 67 to 89% after discharge (Fig. 6c).

## Discussion

Ultrasound-guided technique has been increasingly applied in the management of pain. The obvious advantage is to gain greater precision and safety under the guidance of real-time imaging. To treat neck pain, previous ultrasound-guided method has mainly focused on the regional nerve block therapy, including selective cervical nerve root, cervical facet joints, occipital nerve, and stellate ganglion block^18–21^. In this study, we combined the ultrasound-guided technique with PLD in the treatment of cervical degenerative pain, which has extended the application of ultrasound in the in the field. Our data indicates this novel minimally invasive therapy may be an effective and safe option for those suffer cervical discogenic pain.

To manage cervical pain, the transformation from conservative to surgical intervention remains controversy, due to the lack of high-quality clinical evidence^22^. Consequently, about two thirds of patients reported intolerant pain and incapacity after conservative treatment^23^. According to the clinical guideline in our center, patient may consider PLD when adequate relief from pain cannot be achieved by medication therapy, such as nonsteroid anti-inflammatory drugs, mannitol, and additional analgesic agents. Given the aged population, we think it unnecessary to treat neck conservatively for at least 6 months, due to multiple aged-related factors of disc degeneration^24^.

The lower cervical discs (C5/6 and C6/7) remain the most commonly affected sites in this study, accounting for almost 70% of surgical discs. Currently, neurosurgeon needs to palpate the carotid artery and jugular vein moderately lateral to the cervical spine before inserting the needle. This step seems uncomplicated and straightforward. However, weak or undetected carotid pulsation, obesity, and limited neck motion may hinder the manipulation of cannula. The technical problem following the puncturing may contribute to high risk of complication in the cervical region^11^. Moreover, larger hematoma was reported after repeated puncturing attempts^11^. Thus, novel approach of puncturing technique in PLD is essentially needed to improve the precision and safety of the minimally invasive procedure.

The application of ultrasound in pain management has increased significantly in the last two decades. Compared with traditional blind techniques, ultrasonography provides effective protection of nerve and vessels. In addition to avoid potential risk, ultrasound imaging allows us to place the tip of needle more precisely, and decrease the exposure to the radiation of X-ray. Recently, ultrasound guidance has also been applied in the neuromodulation therapy like pulsed radiofrequency and electrical peripheral nerve stimulation^25,26^. To our knowledge, it is for the first time we report the use of ultrasound-guided in the PLD procedure to treat cervical pain.

The technical note of ultrasound-guided PLD has something in common with selective cervical nerve root block. Comprehensive anatomic knowledge of cervical structures is necessary to perform the surgery. The key step is to recognize the landmark of distinct cervical levels. We recommend to identify the C6 nerve root at initial scan for its characteristic U-type transverse process, as shown in Fig. 2a. Unlike selective nerve root block, the entry point of needle should be relatively medial to the spine to insert into the interverbal disc (Fig. 2c).

Similarly, the C7 level can be identified easily by the straight transverse process, and the blockade of brachial plexus can be performed in this plane. At this localization, the brachial plexus looks like grapes or a cluster of hypoechoic round structures (Fig. 3a), located dorsal and superior to the subclavian artery. Once the tip of needle reaches the anterior surface of vertebral body, the surgeon can slightly move the cannula into the annulus fibrosus without the concerns of vessel injury. After correct placement, the depth of cannulation should be determined by the fluoroscopic imaging (lateral view)^9^. The novel ultrasound-guided technique provides us an effective and safe option for cervical PLD puncturing path, and we did not observe any obvious vessel injury or hematoma in this study.

Consistent with previous data, we found that PLD treatment achieved significant pain relief and functional improvement for cervical discogenic pain^9–11,15^. The overall successful rate reported by previous studies ranged from 75 to 82% according to the Macnab Criteria^11,15^, similarly, we found that the good-to-excellent ratio gradually increased from 67% (1-month follow-up) to 89% at last interview (Fig. 6c). Radiological finding has indicated that laser decompression is not associated progressive cervical degeneration, as demonstrated by the unchanged height of the intervertebral space^27^. Vaporization of herniation provides immediate disc decompression to achieve pain relief, moreover, the shrinkage of collagen may contribute to the latent recovery.

The main limitation of this study is the retrospective and non-comparative nature of design. We think it necessary to conduct randomized, controlled study with larger sample number to compare the therapeutic effect of ultrasound guiding PLD with traditional blind technique, as well as for the complication rate, puncturing attempts, surgical duration, etc.

## Conclusion

The finding of this feasibility assessment indicates the ultrasound-based cannulation technique is capable of guiding the cannulation for the percutaneous laser discectomy. It may facilitate identifying the corresponding site of cervical intervertebral disc and prevent the damage of vessel.

## Data Availability

All data generated or analysed during this study are included in this published article.
